# Preclinical characterization of S095029, a novel clinical-stage Fc-attenuated NKG2A-blocking antibody for cancer immunotherapy

**DOI:** 10.3389/fonc.2026.1924010

**Published:** 2026-09-23

**Authors:** Maria Carlsen Melander, Franziska Uhlenbrock, Anne Worsaae, Randi W. Hansen, Julien Mafille, Daniel Andersen, Samantha Knockaert, Wendy Philippon, Nikolaj Dietrich, Michael M. Grandal, Agnete Marie Granau, Chris D. Madsen, Alba Diaz-Herrero, Fabien Melchiore, Johanna Verneau, Soraia Cerqueira, Matthieu Bouaziz, Mathieu Boucher, Tania Køllgaard, Jamila Elhmouzi-Younes, Niels Jørgen Skartved, Pierre Tonnerre, Céline Lefebvre, Véronique Blanc, Trine Lindsted, Camilla Frölich, Bruno Laugel

**Affiliations:** 1 Servier Symphogen A/S, Ballerup, Denmark; 2 Institut Servier d’Innovation Thérapeutique, Paris-Saclay R&D Center, Rue Francis Perrin, 91190 Gif-sur-Yvette, France; 3 Institut de Recherche Saint-Louis, Université Paris Cité, INSERM U1342, Paris, France; 4 Institut Servier de Médecine Translationnelle, Paris-Saclay R&D Center, Rue Francis Perrin, 91190 Gif-sur-Yvette, France; 5 Keyrus Life Science, Nantes, France

**Keywords:** immune checkpoint inhibition, NKG2A-blocking monoclonal antibody, clinical-stage, immunotherapy, lung cancer, gastric cancer

## Abstract

Natural killer group 2 family of receptor A (NKG2A) and its ligand human leukocyte antigen-E (HLA-E) are increasingly recognised as an important immune checkpoint restraining lymphocyte populations involved in cancer immunity. Recent clinical studies investigating dual NKG2A/programmed death ligand 1 (PD-L1) blockade versus anti-PD-L1 monotherapy suggest that the combination may be beneficial in immunotherapy-responsive disease settings. To date, clinical safety and efficacy results are only available for a single NKG2A-blocking monoclonal antibody (mAb). Here, we report on the generation and preclinical characterization of a novel NKG2A-blocking agent. The fully human Fc-attenuated immunoglobulin G1 mAb S095029, selected via functional antibody repertoire screening, was tested against benchmark clinical-stage antibodies in *in vitro* binding and functional NKG2A/HLA-E blockade assays. S095029 bound human NKG2A with a low nanomolar-range affinity and efficiently reversed the inhibitory effects of NKG2A/HLA-E interaction. As a single agent, S095029 enhanced the activation, cytotoxicity, and cytokine secretion of natural killer (NK) and γδ T cells. S095029 improved NK cell antibody-dependent cellular cytotoxicity and other effector functions triggered by Fc-competent mAbs. *In vivo* investigations in syngeneic mouse models showed that S095029 inhibited tumour growth by harnessing the anti-tumour activity of both NK and CD8+ T cells. Overall, our results support the clinical development of S095029 in immuno-oncology-responsive tumour settings with adequate HLA-E expression. Phase 1b/2 dose escalation studies are currently underway to evaluate S095029 in combination with anti-PD-1 in non-small cell lung cancer and gastric cancer.

## Introduction

Immunotherapy has become a major therapeutic axis in oncology and is now positioned along chemotherapy and targeted therapy for the treatment of cancer patients. PD-(L)1 blockade with monoclonal antibodies (mAbs) has met impressive success in specific solid tumour settings and is now becoming a standard of care in early treatment lines (1, 2). However, despite the wealth of immune checkpoints (ICPs) described in the literature as mechanisms harnessed by tumours to escape immune surveillance, few ICP-targeted therapies have been validated in the clinic beyond PD-(L)1 and CTLA-4 blockade (3).

Natural killer group 2 family of receptor A (NKG2A; CD159a) is a C-type lectin that heterodimerises with CD94 to form an immune suppressive receptor. Upon binding to its ligand, human leukocyte antigen-E (HLA-E), NKG2A transmits inhibitory signals by recruiting the SHP-1 and SHP-2 phosphatases to the Immune Tyrosine-based Inhibition Motifs (ITIMs) of its cytoplasmic tail and regulates the activity of several lymphocytic immune receptors (4, 5).

NKG2A cell surface expression has been documented in natural killer (NK), natural killer T (NKT) and γδ T, innate lymphoid, and cytotoxic T cells, and appears mostly restricted to the blood and lymphocyte-rich tissues at steady-state (6–8). Various cytokines (9–11) and suppressive soluble mediators (12–14) induce the expression of NKG2A. In addition, CD8+ tumour-infiltrating lymphocytes (TILs) in different solid cancers express NKG2A (8, 9, 15–19), suggesting it may affect CD8+ T cell activation at the tumour site. Functional observations have substantiated this hypothesis, showing that NKG2A blockade or knock-out improve the effector functions of CD8+ T cells *in vitro* and *in vivo* (10, 19–22).

The NKG2A/HLA-E axis is part of the natural self-recognition mechanism used by NK and T cells to impart tolerance to self, and maintain immune homeostasis (23–25). However, cancer cells can exploit this pathway by overexpressing HLA-E, which may contribute to evasion of immune surveillance. Indeed, high intra-tumoural expression of NKG2A and HLA-E predicts a poor prognosis for patients with liver and gastric cancers (14, 26, 27). HLA-E can be expressed at high levels in multiple solid and haematological malignancies (28), thus potentially rendering NKG2A/HLA-E blockade broadly therapeutically relevant.

To date, clinical results are available only for the anti-NKG2A antibody monalizumab. The phase 3 clinical trial interim results of monalizumab in combination with cetuximab in head and neck squamous cell carcinoma (HNSCC) patients (NCT04590963) showed no clinical benefit over cetuximab (29). However, monalizumab combined with the PD-L1 blocker durvalumab recently improved objective response rates and progression-free survival compared to durvalumab alone in stage III non-small cell lung cancer (NSCLC) patients post-radiochemotherapy and in a neo-adjuvant setting for patients with resectable tumours (30, 31). These results suggested that NKG2A blockade can complement and improve anti-PD-(L)1 therapy and strengthened interest in NKG2A as an immune checkpoint target with a good safety profile and promising efficacy in adequate clinical settings.

S095029 is a fully human Fc effector function attenuated immunoglobulin G1 (IgG1-LALA) monoclonal antibody that specifically targets human NKG2A, efficiently blocking its interaction with HLA-E. S095029 showed favourable safety and tolerability profiles as a single agent or in combination with a programmed death 1 (PD-1) blocking antibody in a recent first-in-human dose escalation study (32). Here, we report on the generation and selection process, preclinical *in vitro* and *in vivo* functional characterization, and comparison of S095029 with other agents in clinical development. S095029 adds a novel therapeutic agent with discrete properties to the class of NKG2A-blocking antibodies and can contribute to the clinical evaluation of the NKG2A/HLA-E axis as a target for immunotherapy.

## Materials and methods

### Cell lines

Name, supplier, and reference number for each cell line are summarised in **Supplementary Table 1**. All cell lines were mycoplasma free, cultured per supplier recommendations, and continuously evaluated for visual changes. Where relevant, cell lines were transduced using a 3rd generation packaging system made use of four vectors: the lentivector of interest (encoding a trimeric HLA-E (33) or the GD2 and GD3 synthases in tandem with the rat CD2 reporter gene), the packaging (pRSV-REV), envelope (pVSV-G), and accessory plasmids (pMDLg/pRRE). Detailed lentiviral production procedures are described in Li et al. (34).

### Flow cytometry

Details on the antibodies used for *in vitro* and *in vivo* functional, phenotypic and structural investigations are summarised in **Supplementary Table 2**. To test antibody candidates for their ability to block phycoerythrin (PE)-conjugated HLA-E pentamer (F712-2C_E, Proimmune) binding to NIH3T3 cells expressing hNKG2A/hCD94, antibodies were added to cells and incubated for 20 minutes at 4˚C. After washing, HLA-E pentamer was added to the cells at a 1:50 final dilution and incubated for 20 minutes at 4˚C in the dark. Cells were acquired on an iQue screener flow cytometer to measure binding. Antibody concentrations ranged from 30 µg/ml to 14 ng/ml (three-fold dilutions).

Epitope binding analyses were performed with CHO cells expressing CD94 with wild-type human NKG2A or NKG2A mutants AA substitutions: S167A, I168S, S170L, M189I, E197K, I225M). The antibodies, diluted three-fold from 30 ug/ml to 0.5 ng/ml, were added to the cells and incubated for 30 minutes at 4˚C. Cells were washed and incubated with fluorescent secondary antibody goat anti-human IgG (H+L) AF647 (A-21445, Invitrogen).

To phenotype patient samples, frozen NSCLC and gastric cancer samples were thawed and processed using standard surface staining protocols. All samples were acquired on a CytoFlex LX instrument (22-colors, Beckman Coulter). Data were analysed using FlowJo software (BD Biosciences). The primary antibodies used were: CD3, CD4, CD8, CD16, CD39, CD45, CD56, CD73, EpCAM, HLA-E, LILRB1, NKG2A, PD-1, PD-L1, TCR γδ, and TIM-3 (**Supplementary Table 2**). Fluorescence-minus-one controls served to establish fixed thresholds for NKG2A and PD-1.

### NKG2A receptor quantification on primary cells

PBMC samples were thawed, washed twice with Roswell Park Memorial Institute (RPMI) medium +10% foetal calf serum (FCS) +1% penicillin-streptomycin. Anti-NKG2A (clone REA 110, Miltenyi) antibody binding capacity was determined using saturating concentration and the BD Biosciences QuantiBRITE (BD Biosciences) system. To quantify NKG2A expression on γδ T cells and NK cell subsets, PBMC samples were stained with a panel of antibodies consisting of anti- γδ TCR, anti-CD3, anti-CD56, and anti-CD16 in addition to the anti-NKG2A antibody. QuantiBRITE PE beads (BD Biosciences) were acquired on a CytoFlex LX instrument on the same day and at the same instrument settings as the individual PBMC samples. A standard curve comparing the geometric mean of fluorescence to known PE content of the QuantiBRITE beads was constructed. The regression analysis, slope, y-intercept and correlation coefficient were determined. Antibody binding capacity values were generated from the measured geometric mean fluorescence of NKG2A on γδ T cells and NK cell subsets, based on γδ TCR/CD3 co-expression and CD3 negative/CD56 positive stainings, respectively, using the QuantiBRITE standard curve.

### *In vivo* assays

Mice were maintained under standard housing conditions and allowed to acclimatise for one week prior to the initiation of experimental procedures. For all procedures, animals were anesthetised with isoflurane inhalation anaesthesia and at the end of the study, mice were euthanised by cervical dislocation in accordance with Danish regulations for humane animal care and use. The animal studies reported in this article were designed, conducted and analysed according to the ARRIVE guidelines.

To analyse *in vivo* efficacy of S095029 alone or in combination with the anti-programmed cell death protein 1 (PD-1) SYM021, 2x10^6^ murine MCA205 fibrosarcoma or 2x10^6^ murine MC38 colon carcinoma cells transduced with human HLA-E were subcutaneously (s.c.) engrafted onto human NKG2A/CD94 double knock-in mice (females., 5–8 weeks, Biocytogen, China). Tumours were measured three times weekly by caliper in two dimensions, and tumour volume (mm^3^) was calculated per the formula: (width)^2^ x length x 0.5. Treatment was initiated at an average tumour volume of 50–70 mm^3^ and the mice were treated three times weekly by intraperitoneal (i.p.) injection of vehicle buffer, S095029 (10 mg/kg), the SYM021 anti-PD-1 (0.5–1 mg/kg), or a combination of S095029 and SYM0211 (n=10/group, approximately 10% of the total number of mice included in the experiment were removed at randomization based on outlier tumour volumes).

For NK and CD8 depletion studies, mice were treated by i.p. injection of 0.2 mg of anti-NK1.1 mAb clone PK136 (BioXcell Cat # BP0036) or anti-CD8a mAb clone YTS 169.4 (BioXcell Cat # BP0117) twice weekly for four doses (n=10/group). Depletion was initiated at the same time as antibody treatment and was verified by flow cytometer analysis of whole blood cells at study day 15 as described under section flow cytometry. For verification of *in vivo* CD8 and NK cell depletion, tail vein blood was sampled in ethylenediaminetetraacetic acid (EDTA) and lysed for red blood cells (RBC) using RBC lysis buffer (Biolegend cat# 420301). After adding murine Fc block (BD Pharmingen, 553142) and dead cell exclusion marker (BD Pharmingen, L34976), cells were stained for cell surface markers using the following mAbs: CD335-BV421, CD8-BV605, CD4-BUV395, CD3-BUV496, CD45-BUV805 and TER119-APC-Cy7. Cells were acquired using FACSymphony A3 Cell Analyzer and were analysed using FlowJo Software version 10.9.0.

For the experimental lung metastasis mouse model, human NKG2A/CD94 double knock-in mice (females., 5–8 weeks, Biocytogen, China) were treated with a single dose of vehicle buffer or 10 mg/kg S095029–1 hour before intravenous (i.v.) injection of either 1x10^6^ B16F10 melanoma tumour cells (ATCC-CRL-6475) stably transduced with human HLA-E trimer and red-shifted firefly luciferase (IVISbrite Red F-Luc-GFP, Revvity, #CLS960003) or B16F10 tumour cells transduced only with luciferase (n=3-4/group, n=19 in total). For bioluminescent quantification of tumour growth, mice were i.p. injected with 150 mg/kg D-luciferin potassium (IVISbrite Revvity #122799) and analysed by whole animal optical imaging using an Ami HT small animal imager (Spectral Instruments Imaging). Images were acquired after 1, 7 and 24 hours, and on days 2, 3, 4, 5, 7, 9, and 11.

For treatment and depletion experiments, mice were treated i.p. with vehicle buffer or 10 mg/kg S095029 and anti-NK1.1 mAb clone PK136 (BioXcell Cat # BP0036) at 0.2mg/mouse twice weekly prior to tumour cell injection for a total of two treatments (n=5-7/group, n=28 in total). An additional treatment with S095029 was given 1 hour before tumour cell inoculation. Bioluminescence images were acquired after 1 hour, once daily until day 4, and on days 7 and 8. Two days after tumour inoculation, depletion was verified by flow cytometer analysis of whole blood cells, as described above. Mice were excluded from analysis if tumour cells were incorrectly administered, there were technical issues during imaging or insufficient administration of D-luficerin.

### Calcein-based NK cell killing assays

Target cancer cell lines including (K562 over-expressing HLA-E, HT-29, CCRF-CEM, A253, Detroit 562, CAL-120 or FaDu) cells were pulsed overnight with HLA-B*0701 leader sequence peptide (VMAPRTVLL) and NK92 cells were interleukin (IL)-2 starved overnight. The next day, NK cells were isolated from healthy donors; anti-NKG2A antibodies were incubated at various concentrations with NK92 or purified primary NK cells, were added to calcein-loaded target cells at effector-to-target ratios of 10:1, and were incubated for 90 minutes. The killing capacity of NK92 or primary NK cells was measured by calcein release in the supernatants. Specific lysis was calculated by subtracting spontaneous lysis (calcein-loaded K562-HLAE cells only) and normalizing to maximum lysis (Triton X-100 lysis of calcein-loaded K562-HLAE cells).

### Cytokine release assays

Target cancer lines stably transduced with a lentiviral vector overexpressing HLA-E (JIMT-1, SKOV-3, BxPC3 and A549) were cocultured with NKG2A+ NK cells isolated from fresh PBMCs at a 1:10 ratio in the presence of 10 ng/ml IL-2, anti-HER2 (trastuzumab analogue), and anti-NKG2A. After 48 hours, supernatants were collected, and the concentration of macrophage inflammatory protein (MIP)-1β was quantified by ELISA (Invitrogen, 88-7034-88). Alternatively, peripheral blood lymphocytes (PBLs) from healthy donors were isolated by depleting CD14+ cells from PBMCs. 50,000 PBLs were plated in round-bottom 96-well plates with 5,000 SKOV3 cells transduced with lentiviral vectors overexpressing HLA-E and activated with trastuzumab 1 µg/ml. In addition, PBLs were treated with S095029 (10 µg/ml) or with the corresponding isotype control IgG1-LALA (10 µg/ml). After 3 days of incubation, concentrations of soluble mediators in supernatants were determined by electrochemoluminescence with a multi-cytokine panel U-Plex kit (K151AEL-4, Mesoscale Discovery) on a Meso QuickPlex SQ120 plate reader (Mesoscale Discovery). Levels of MIP-1α (BD; Cat# 558325), granzyme B (BD; Cat# 560304), IFN-γ (BD; Cat# 558269) and tumour necrosis factor (TNF) (BD; Cat# 558273) were quantified by bead array on a LSR Fortessa (BD) cytometer. Subsequent analysis of cytokine concentrations was carried out with the software FCAP Array v 3.0 (BD; Cat# 652099).

Cytokines from co-culture supernatants between NK cells expressing NKG2A (effector cells; three donors) and K562 or A431 (target cells) were determined using three different Luminex kits (human immune-oncology checkpoint protein panel 2, human cytokine/chemokine panel 1, human CD8+ T cell panel; Merck Millipore) per manufacturer’s instructions. Data were generated by the Luminex^®^ BioPlex manager.

Supernatants from co-cultures of NK and target cells were collected after 48 hours and frozen. IFN-γ, MIP1-α, and MIP1-β concentrations were then measured by Meso Scale Discovery (MSD) multiplex immunoassay (U-PLEX technology) as per manufacturer’s instructions.

For each cytokine measurement in supernatants, XY data tables were compiled with anti-NKG2A antibody concentration (X) and cytokine concentration (Y). X values were then transformed to logarithmical scale (X = LogX) and a nonlinear regression (curve fit) analysis was applied with a constant equal to the highest concentration measured as top constraint. Data are presented as means plus/minus SD.

### Animal studies ethics approval

All *in vivo* studies were carried out in Denmark, ethically approved and performed in accordance with applicable laws and regulations relating to the care and use of laboratory animals and in compliance with Good Laboratory Practice (GLP). All animal experiments were approved by the Danish Animal Experiments Inspectorate (DAEI, licence numbers 2021-15-0201–00899 and 2021-15-0201-01184) and were performed in accordance with institutional and national guidelines for animal care and use. All the *in vivo* studies reported in this article were performed between March 2020 and February 2025.

### Human samples ethics approval

The studies involving healthy human donor samples were approved by the ethics committee of Etablissement Français du Sang (EFS), Ile-de-France (agreement n°21/EFS/035). The studies were conducted in accordance with the local legislation and institutional requirements. The participants provided their written informed consent to participate in this study. Patients recruited at the Bichat and Saint-Louis hospitals were included according to the criteria of the approved ethical protocol CLEA-2020–113 and signed an informed consent for their participation. The collection protocol(s) and informed consents (when required) used by Discovery Life Sciences (Discovery) to obtain the accompanying human biospecimens as part of the study protocols 2B_BB044, 2F_SB_GP, 2H_SB_GP, 3K_BB044, 4E_BB044, 6D_BB044, 6D_SB_GP, G9_198, H1_ABRT, Z1_BB041 have been approved by an Institutional Review Board (IRB)/Ethics Committee (EC) in accordance with applicable country-specific regulatory requirements. The studies were conducted in accordance with the International Conference on Harmonization (ICH) Good Clinical Practice (GCP) guidelines and to the ethical principles outlined in the Declaration of Helsinki 2013, including all patient privacy requirements. Patient samples used in for the work reported in this article were collected between August 2019 and February 2024.

### Statistical analyses

All statistical analyses were performed with the R software version 4.4.1 using statistical tests specified in relevant figure legends.

## Results

### Generation of NKG2A-blocking antibodies and selection of the S095029 candidate

A repertoire of NKG2A-binding antibodies was generated by OmniRats^®^ immunization and single-cell sorting of lymph node-isolated plasma cells. The corresponding antibody library was expressed in a full-length IgG1-LALA heavy chain format and screened for binding to human and cynomolgus NKG2A as well as human natural killer group 2 member C (NKG2C) by high throughput flow cytometry of raw plasma cellular supernatants (**Figure 1A**). 738 antibody binding hits with unique sequences and no apparent developability issues, as determined by sequence analyses, were produced, purified in miniscale format, and taken forward for further hNKG2A/hNKG2C/cyNKG2A binding tests (data not shown). 379 candidates selected from their binding profiles were taken forward for functional testing in NK cell cytotoxicity assays. Six of these were chosen based on their capacity to enhance the killing activity of NK cells (**Figure 1B**). Following dose-response binding analyses to cognate ligands (**Supplementary Figure 1**) and HLA-E blockade experiments (**Supplementary Figure 2**) comparing the six candidates to each other and to a monalizumab analogue, the S095029 candidate (corresponding to clone 2420) was selected for further characterization and development.

**Figure 1:**
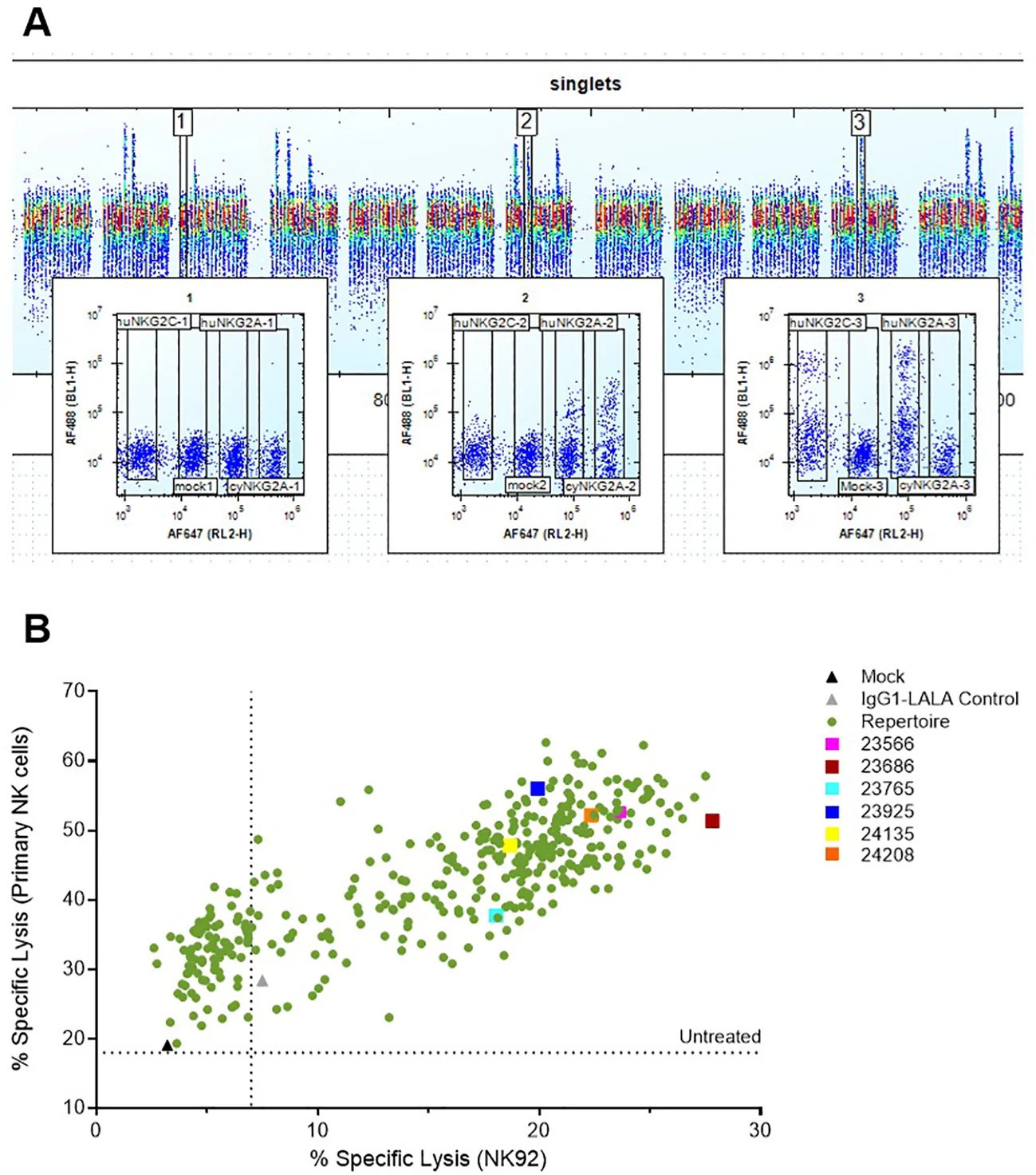
Screening and selection of lead anti-NKG2A antibody candidates. **(A)** High-throughput antibody staining of CHO-S cells transfected with plasmids containing DNA coding for hNKG2A/hCD94, cNKG2A/cCD94, and hNKG2C/hCD94. Inset 1: screening result from a single well in a 384-well plate showing an antibody with no binding to any of the targets. Inset 2: well containing an antibody binding to both hNKG2A/hCD94 and cNKG2A/cCD94 and a possible weak response to huNKG2C/hCD94. Inset 3: well containing an antibody specific for hNKG2A/hCD94 and hNKG2C/hCD94. **(B)** Correlation of NK-92 cell line and primary NK cell killing enhancement of K562/HLA-E by anti-NKG2A antibodies (25 µg/ml). Dots indicate the average value of triplicates. The dotted lines indicate levels of killing in samples not treated with antibodies. c, cynomolgus; CHO-S, Chinese hamster ovary; h, human; HLA-E, human leukocyte antigen-E; NKG2A, natural killer group 2 family of receptor A; NKG2C, natural killer group 2 member C; NK-92, natural killer-92.

### Binding properties of the S095029 candidate

S095029 bound the NKG2A/CD94 extra-cellular domains with a K_D_ of 2.6 nM (**Table 1**) and strongly stained cells expressing the human CD94/NKG2A heterodimer on their surface, but not cells expressing human NKG2C or cynomolgus NKG2A or NKG2C complexes in flow cytometry studies (**Figure 2A**). S095029 selectively disrupted HLA-E pentamer binding to the NKG2A/CD94 complex in antibody blockade experiments with little inhibition of HLA-E binding to NKG2C/CD94 heterodimers (**Figure 2B**).

**Table 1:** Biophysical binding properties for the S095029 and a monalizumab analogue determined by surface plasmon resonance.

mAb	ECD	ka ± SD (M-1s-1)	kd ± SD (s-1)	KD ± SD (nM)
S095029	HuNKG2A/huCD94	5.9E+05 ± 6E + 04	1.5E-03 ± 3E-05	2.6 ± 0.3
	HuNKG2C/huCD94	1.0E+04 ± 1E + 03	1.2E-03 ± 3E-05	123 ± 11
Monalizumab	HuNKG2A/huCD94	4.4E+05 ± 5E + 04	5.4E-03 ± 4E-04	12.5 ± 0.5
	HuNKG2C/huCD94	6.5E+03 ± 5 E + 02	4.4E-03 ± 3E-04	682 ± 34

On-rate (k_a_), off-rate (k_d_), and affinity (K_D_) constants are shown for extracellular domains (ECDs) of different antigens binding to immobilised antibodies (mAbs). The corresponding surface plasmon resonance (SPR) traces are shown in **Supplementary Figure 3**. hu, human; NKG2A, natural killer group 2 family of receptor A; NKG2C, natural killer group 2 member C; SD, standard deviation.

**Figure 2:**
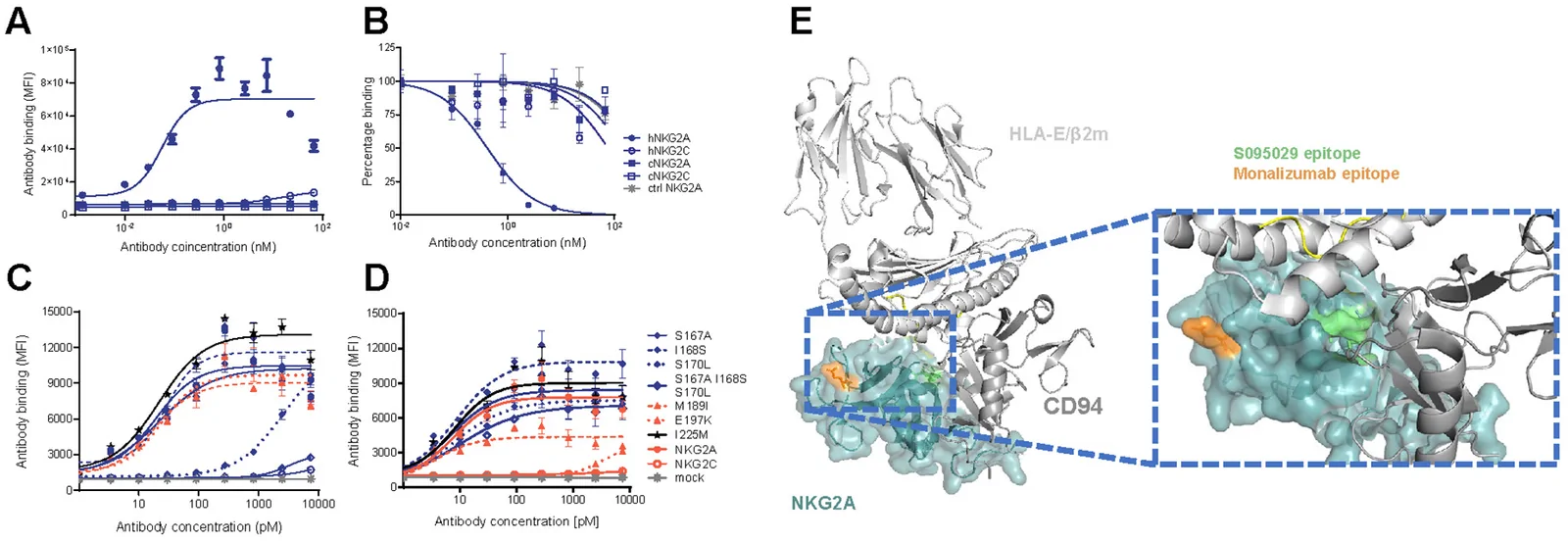
Binding properties of S095029 on cell surface NKG2A/CD94 complexes. **(A)** S095029, added in three-fold serial dilutions from a 3 µg/ml starting concentration, was tested for binding to CHO-S cells transiently transfected with hNKG2A/cCD94, cNKG2A/cCD94, hNKG2C/hCD94 and cNKG2C/cCD94. A secondary AF647 fluorochrome-labelled a-human IgG(H+L) was used as detection antibody. Background binding was determined with an isotype control. **(B)** Blocking of HLA-E pentamer binding by the S095029 antibody in serial dilutions prior to HLA-E pentamer staining to CHO-S cells transiently transfected with hNKG2A/cCD94, cNKG2A/cCD94, hNKG2C/hCD94 and cNKG2C/cCD94, as indicated. S095029 **(C)** and monalizumab analogue **(D)** epitope mapping by flow cytometry using CHO-S cells transiently transfected with WT CD94 and mutated NKG2A bearing substitutions at the AA residues diverging between NKG2A and NKG2C (NKG2A positions 167, 168, 170, 189, 197, and 225), as indicated. Antibodies were tested at 10 µg/ml and three-fold dilutions thereof. **(E)** Key residues involved in mAb binding, as determined in **(C)** and **(D)**, mapped on the structure of NKG2A/CD94 in complex with HLA-E/β2 are represented as sticks and colored green for S095029 (S167, I168, and S170) or orange for the monalizumab analogue (E197). For visualization and structural interpretation, the amino acids of interest were highlighted in the published structure of human CD94/NKG2A in complex with HLA-E (PDB: 3CDG) using PyMol. c, cynomolgus; CHO-S, Chinese hamster ovary; h, human; HLA-E, human leukocyte antigen-E; IgG, immunoglobulin G; MFI, mean fluorescence intensity; NKG2A, natural killer group 2 family of receptor A; NKG2C, natural killer group 2 member C.

In a targeted mutagenesis approach focusing on the sixAA residues diverging between the extra-cellular domains of NKG2A and NKG2C, a triple substitution at residues 167, 168, and 170 was required to abrogate S095029 binding to the NKG2A/CD94 complex (**Figure 2C**). In contrast, binding of a monalizumab analogue to NKG2A was abrogated by single substitutions at positions 189 and 197 (**Figure 2D**). When mapped to the crystal structure of NKG2A/CD94 in complex with HLA-E, the S095029 binding epitope appeared to be located close to the NKG2A/CD94 binding interface next to the P172 residue in direct contact with HLA-E (**Figure 2E**). Monalizumab binding, however, appeared highly dependent on residue 197E, which protrudes from the NKG2A surface away from the NKG2A/HLA-E interaction interface (**Figure 2E**).

### S095029 functional characterization on cell lines and primary immune cells

We confirmed that NKG2A is expressed on the surface of most NK and γ9δ2 T cells in healthy human blood cells, whilst few peripheral CD8+ T cells are NKG2A+ (**Figure 3A**). The average density of NKG2A receptors in these cell populations was 3,000 to 6,000 molecules per cell (**Figure 3B**), with γδ T cells and CD16^neg^ NK cells expressing higher receptor numbers. Flow cytometry staining of NKG2A+ primary human cells with S095029 identified NK and γδ T cell proportions in agreement with standard donor NKG2A positivity within these populations, and a binding efficacy to primary cells comparable to cell lines overexpressing the CD94/NKG2A complex (**Figures 2A**, **3A, C, D**).

**Figure 3:**
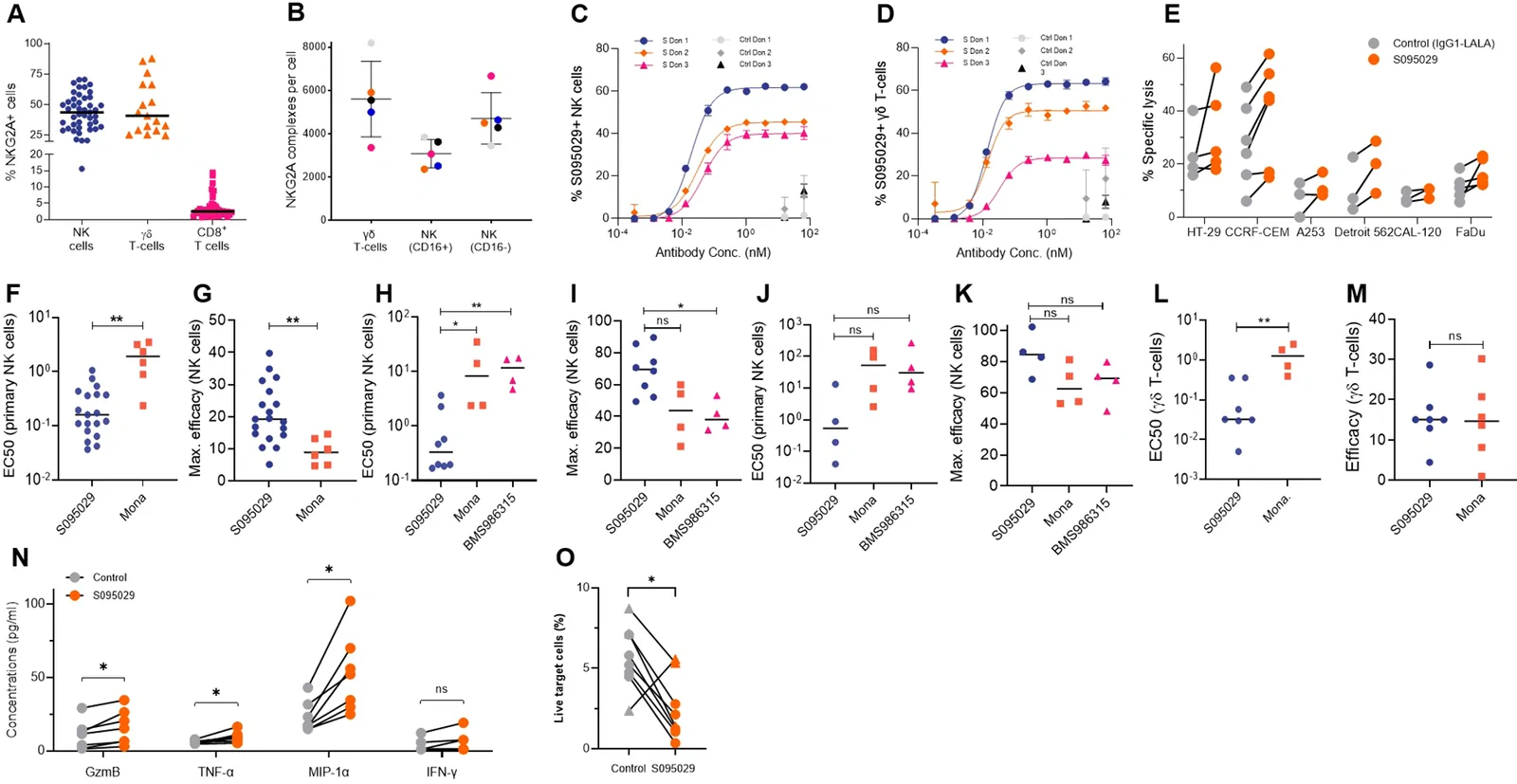
Enhancement of direct NK cell activation by tumour cells with NKG2A-blocking antibodies. **(A)** Expression of NKG2A on the NK cells, γδ T cells, and CD8 T cells from healthy individuals. Each dot represents a donor. The NK and CD8+ T cells of 45 donors were stained for NKG2A, and the γδ T cells of 17 donors were phenotyped. Median values were 43.7%, 71.4%, and 2.5% for NK, γδ T cells, and CD8+ T cells, respectively. **(B)** Quantification of NKG2A receptor cell-surface density in NKG2A+ cell types by flow cytometry using peripheral blood lymphocytes isolated from five healthy donors. The different cell populations were identified by differential gating on the live, CD3, γδ TCR, and CD56 markers. **(C)** Dose-response S095029 binding to NK cells. Coloured dots represent samples stained with S095029 at the indicated concentrations, whilst grey and black dots represent isotype control staining performed at 16.5 and 66 nM. Binding EC_50_ values on NK cells were 19.40, 31.76, and 47.31 pM for donors 1, 2 and 3, respectively. **(D)** Dose-response S095029 binding to γδ T cells isolated from three healthy donors. Binding EC_50_ values on γδ T cells were 13.63, 14.4, and 33.83 pM for donors 1, 2 and 3, respectively. **(E)** Freshly-isolated primary NKG2A^+^ NK cells derived from healthy humans were co-cultured with six different, calcein-labelled tumour cell lines (HT-29, CCRF-CEM, A253, Detroit 562, CAL-120, and FaDu) that express endogenous levels of HLA-E and loaded overnight with the HLA-B*0701 signal peptide (U989CGA290-1, GenScript) at 50 µM. Cells were treated with a single dose of S095029 or mAb control. Release of calcein was measured after approximately 3 hours, and percent-specific lysis was calculated. EC_50_ expressed in µg/ml **(F)** and maximum activity **(G)** values of K562/HLA-E+ target cell killing enhancement by S095029 or a monalizumab analogue antibody using primary NK cells freshly isolated from 19 and six healthy donors as effectors, respectively. EC_50_ expressed in µg/ml **(H)** and maximum activity **(I)** values of A375/HLA-E+ target cell killing enhancement by S095029, BMS986315, or monalizumab antibody analogues using *ex vivo* expanded primary NK cells isolated from healthy donors. Each dot represents a donor. EC_50_ expressed in µg/ml **(J)** and maximum activity **(K)** values of SKOV-3/HLA-E+ target cell killing enhancement by S095029, BMS986315 or monalizumab antibody analogues using *ex vivo* expanded primary NK cells isolated from healthy donors. EC_50_ expressed in µg/ml **(L)** and maximum activity **(M)** values of K562/HLA-E+ target cell killing enhancement by S095029 and monalizumab analogue antibodies with primary γδ T cells. The line indicates median values **(F–M, N)** Increase in cytokine secretion by healthy donor NK cells co-cultured with SUDHL6 target cells in the absence or presence of S095029 at 5 µg/ml. Cytokine concentrations were measured with cytometric bead array. Wilcoxon signed rank test was applied for two-group comparisons of paired and non-paired nonparametric data. **(O)** Increase in the killing activity of NK cells isolated from follicular lymphoma (triangles) and diffuse large B-cell lymphoma (circles) patient peripheral blood mononuclear cells against the SUDHL-6 cell line upon treatment with S095029. Non-parametric Wilcoxon signed rank test was applied for two-group comparisons of paired **(N, O)** and non-paired **(E–M)** data: ns (not significant) >0.05, *<0.05, **<0.01). Statistical analyses were performed using the R software version 4.4.1 and adjusted for multiple comparisons. EC_50_, half maximal effective concentration; HLA-E, human leukocyte antigen-E; IgG, immunoglobulin G; NK, natural killer; NKG2A, natural killer group 2 family of receptor A; max., maximum.

Next, we examined the capacity of S095029 to reverse NKG2A-mediated inhibition of NK cell cytotoxicity in co-cultures with tumour cells. NKG2A blockade enhanced direct NK cell cytotoxicity against cancer cell lines of different tissue origin with varying efficiency (**Figure 3E**). In co-culture systems of primary NK cells and tumour cells, treatment with S095029 showed consistently higher potencies and maximum activities as compared to monalizumab and BMS936315. This was consistent across three different cell line models with endogenous (K562) or ectopic (A375 and SKOV3) HLA-E expression (**Figures 3F–K**; **Supplementary Figures 4**, **S5**). We obtained similar results in direct γ9δ2 T cell cytotoxicity experiments using K562 cells as targets (**Figures 3L, M**). When co-cultured with the diffuse large B-cell lymphoma cell line (SUDHL6 DLBCL) expressing endogenous levels of HLA-E (**Figure 3N**), NK cells from healthy donors secreted higher levels of granzyme B, TNF-α, and MIP-1α whilst the levels of IFN-γ were unchanged. Finally, the killing activity of NK cells isolated from the blood of DLBCL and follicular lymphoma patients was improved in presence of S095029 (**Figure 3O**).

These results demonstrate that S095029 efficiently blocks the inhibitory activity of NKG2A in primary NK and γ9δ2 T cells, resulting in enhanced killing of HLA-E-positive tumour cells. Similar observations were made when NK cells were activated by the antibody-dependent cellular cytotoxicity (ADCC)-inducing antibodies cetuximab and dinutuximab: S095029 showed high potencies in killing assays compared with monalizumab and BMS986315 antibody analogues (**Supplementary Figures 6A–E**, **S8**).

### S095029-mediated NKG2A blockade selectively induces the upregulation of soluble factors by NK cells

A mini-screen, performed to assess the effects of S095029-mediated NKG2A blockade on other NK cell effector functions, showed that NK cells secreted higher amounts of several cytokines upon activation by different cell lines. Interestingly, all cytokines produced by NK cells were not affected to the same extent by the addition of S095029 (**Figures 4A–D**), suggesting differential effects on distinct effector functions. The two consistently most-upregulated cytokines in the presence of S095029 were MIP-1α and MIP-1β (three- to four-fold increase compared to isotype control), whereas IL-2, granzyme, and perforin secretion were not substantially improved by NKG2A blockade.

**Figure 4:**
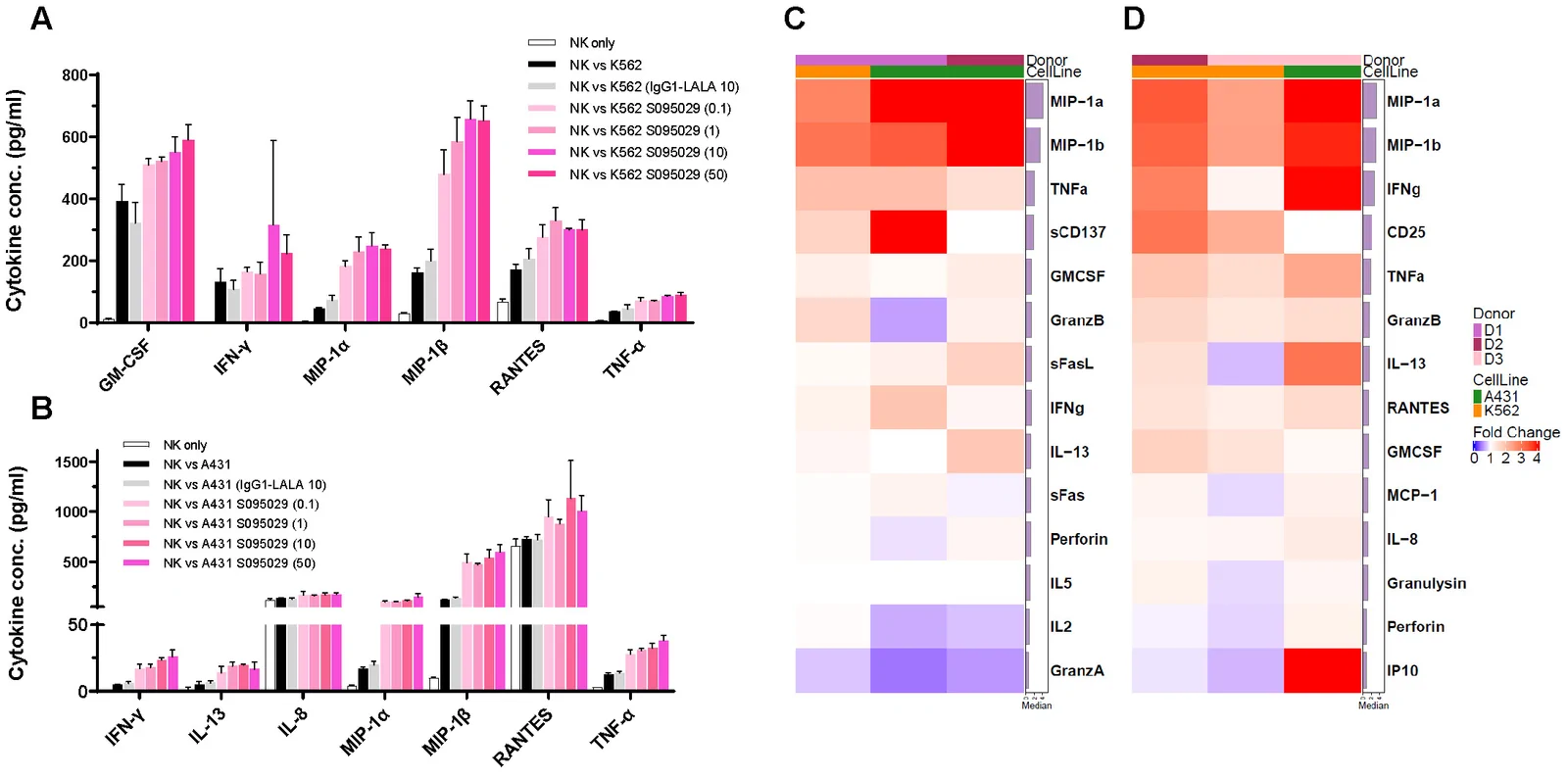
S095029-mediated modulation of soluble factors secretion by NK cells. S095029-mediated increase in cytokine secretion by primary human NK cells co-cultured with K562 **(A)** or A431 tumour cells **(B)** in the absence of antibodies or in the presence of isotype control antibody at 10 µg/ml or with increasing concentrations of S095029, as indicated in the image key. **(C)** Heat map modulation profile summary of 20 cytokines and soluble mediators secreted by NK cells upon NKG2A blockade. **(D)** Heat map representation of an array of soluble factors secreted by NK cells isolated from three donors in co-culture with K562 or A439 target cells, as indicated by the colour code. The heat map colours represent cytokine concentration fold change when calculating the concentration ratio of the 10 µg/ml S095029 condition to the 10 µg/ml isotype control (IgG1-LALA) for NK cells from 3 donors co-cultured with either cell line, as indicated in the map legend. conc., concentration; GMCSF, granulocyte-macrophage colony-stimulating factor; GranzA, granzyme A; GranzB, granzyme B; HLA-E, human leukocyte antigen-E; IFN, interferon; IgG1, immunoglobulin G1; IL, interleukin; MIP, macrophage inflammatory protein; NK, natural killer; NKG2A, natural killer group 2 family of receptor A; max., maximum; sCD137, soluble CD137; sFasL, soluble Fas ligand; RANTES, Regulated upon Activation, Normal T cell Expressed and Secreted; TNF, tumour necrosis factor.

We next compared the effects of different NKG2A blocking antibodies on three NKG2A-dependent cytokines. In agreement with the cytotoxicity read-out, the secretion of IFN-γ, MIP-1α, and MIP-1β was consistently enhanced with greater sensitivity and maximum cytokine concentrations when treated with S095029 as compared to monalizumab or BMS986315 (**Supplementary Figures 3, 5**). This effect was consistent across several donors.

### Combination of S095029 and anti-PD-1 reduces primary tumour growth in a CD8+ T cell-dependent manner

In syngeneic hNKG2A/hCD94 double knock-in mouse model using the s.c. implanted HLA-E-transduced MCA205 fibrosarcoma or the MC38 colon carcinoma tumour cell lines, S095029 single treatment effects varied between experiments, ranging from limited to significant tumour growth reduction (**Figures 5A–D**); whereas anti-PD-1 single treatment consistently reduced tumour growth. The combination of S095029 with anti-PD-1 robustly enhanced anti-tumour activity compared to single treatments.

**Figure 5:**
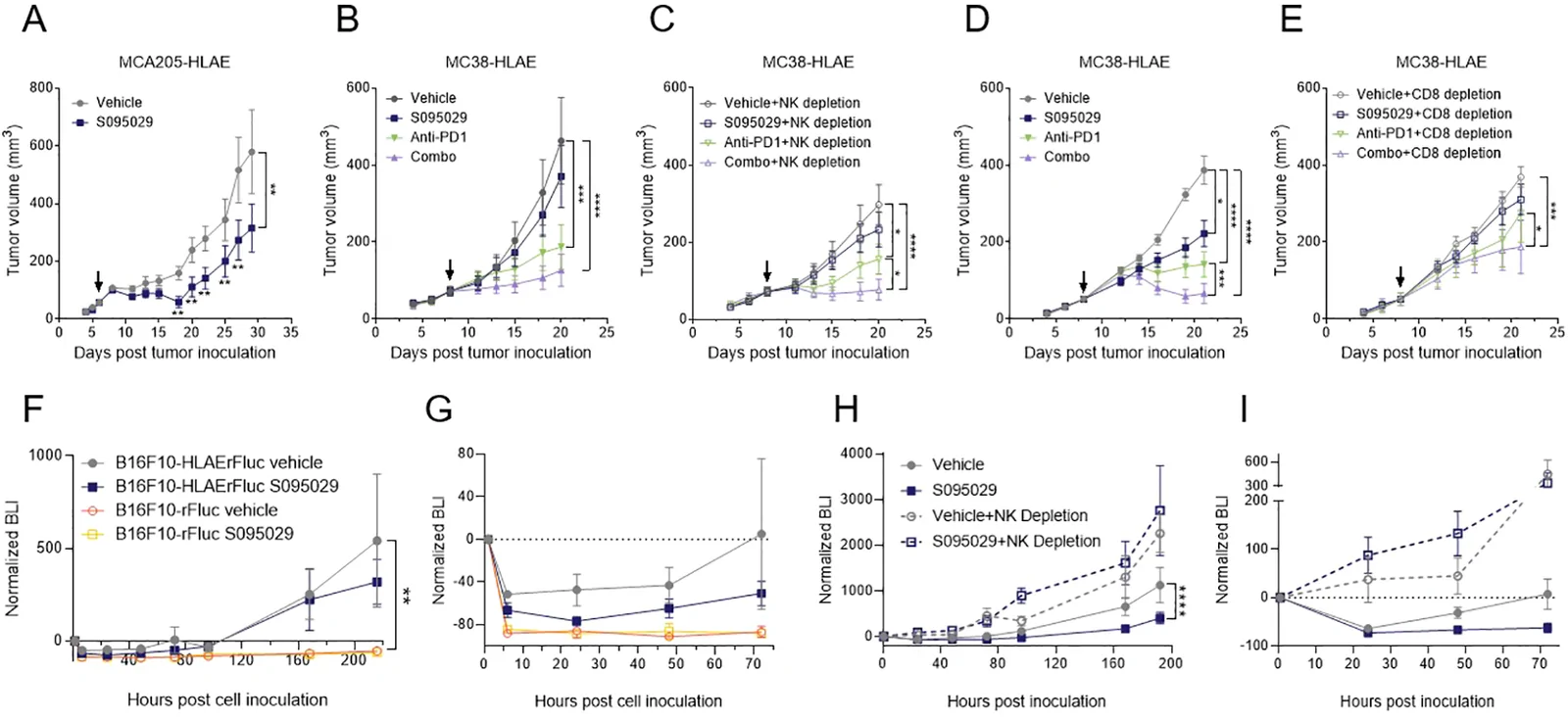
*In vivo* characterization of S095029, alone or in combination with anti-PD-1, in CD94/NKG2A-humanised syngeneic mouse models. **(A)** MCA205 tumour cells transduced with human HLA-E were engrafted subcutaneously in hNKG2A/hCD94 knock-in mice. After randomization, the mice were treated three times weekly with single treatment of S095029 dosed at 10 mg/kg. **(B, D)** MC38 tumour cells transduced with human HLA-E were engrafted subcutaneously in hNKG2A/hCD94 knock-in mice. After randomization, the mice were treated three times weekly with a single S095029 dose at 10 mg/kg, the SYM021 anti-PD-1 mAb dosed at 1 mg/kg, or a combination of both and/or 0.2 mg NK **(C)** or CD8 **(E)** depleting mAbs twice weekly for a total of four doses. Tumour volume was measured three times weekly. Arrows denote treatment start; data are presented as means ± SEM. **(F)** B16F10 tumour cells transduced with human HLA-E and/or luciferase were injected in the tail vein 1 hour after single-dose treatment of vehicle buffer or S095029 at 10 mg/kg. Metastatic tumour burden in the lungs was visualised and quantified using a bioluminescence imaging system from 1 hour to 9 days after tumour cell inoculation. **(G)** Plot focusing on timepoints 1 to 72 hours after tumour inoculation. **(H)** B16F10 tumour cells transduced with human HLA-E and luciferase were injected in the tail vein after pre-treatment twice weekly for a total of 2 doses. with vehicle buffer or S095029 at 10 mg/kg and NK depletion mAb at 0.2 mg/mouse. An additional treatment with S095029 was administered 1 hour before tumour cell inoculation. Metastatic tumour burden in the lungs was visualised and quantified using a bioluminescence imaging system from 1 hour to 8 days after tumour cell inoculation. **(I)** Plot focusing on timepoints 1 to 72 hours after tumour inoculation. Statistical analyses were performed using the R software version 4.4.1. Tumour volume measurements of individual mice for the NK antibody and CD8 T-cell depletion studies are shown in **Supplementary Figure 9**. BLI, bioluminescence imaging; combo, combination; HLA-E, human leukocyte antigen-E; hNKG2A, human natural killer group 2 family of receptor A; mAb, monoclonal antibody; NK, natural killer; PD-1, programmed cell death protein 1.

Upon depletion of NK cells, the anti-tumour effect was neither affected in the S095029 single treatment group nor in combination with anti-PD-1, indicating that NK cells were not the main drivers of the anti-tumour activity (**Figures 5B, C**). In contrast, upon depletion of CD8+ T cells, tumour growth inhibition was reduced, but not completely abrogated, in all treatment groups (**Figures 5D, E**), suggesting that CD8+ T-cells are the main cell type involved in the anti-NKG2A/PD-1 anti-tumour response.

### Blocking NKG2A/HLA-E axis with S095029 reduces early colonization of lung metastasis in a NK-dependent manner

In the experimental lung metastasis model, bioluminescent imaging of lungs from animals injected with B16F10-HLAE tumour cells in the tail vein showed greater tumour burden than mice injected with non-HLAE expressing B16F10 tumour cells; when mice were treated with S095029 (single dose of 10 mg/kg), tumour growth was reduced (**Figures 5F, G**).

In mice treated with S095029 (10 mg/kg) and anti-NK1.1 depletion mAb (0.2mg/mouse) before tumour cell injection, metastatic growth was significantly increased compared to non-NK-depleted mice, and treatment with S095029 had no effect (**Figures 5H, I**).

### Expression of NKG2A and PD-1 on cancer patient samples

To generate additional translational data relevant to S095029’s clinical development, we phenotyped dissociated tumour cells obtained from 19 NSCLC patients and 17 gastric cancer patients. In all patient samples, a sizeable proportion of CD8+ T cells and NK cells expressed NKG2A with an important inter-patient variability (**Figures 6A, E**). The frequency of NKG2A positivity was substantially higher for CD8+ T cells isolated from patient tumours than for CD8+ T cells isolated from the blood of healthy donors (**Figure 3A**). The proportions of NKG2A+ NK cells at tumour sites were in the range of those of blood NK cells (**Figures 3A, C**). PD-1+ cells were often found amongst CD8+ TILs, whereas only few NK cells expressed PD-1 (**Figures 6B, F**). CD8+ T cells constituted the majority of all NKG2A+ TILs for both NSCLC (**Figure 6C**) and gastric cancer (**Figure 6G**) samples, averaging 57% and 51%, respectively. In contrast, NK cells represented between 11% (gastric) and 13% (NSCLC) of NKG2A+ cells on average. Interestingly, most, but not all, NKG2A+ CD8+ TILs also expressed PD-1; 65% of NSCLC and 61% of gastric sample CD8+ TILs exhibited a NKG2A+/PD-1+ double positive phenotype (**Figures 6D, H**), leaving approximately a third as single positive NKG2A+/PD-1- CD8+ T cells.

**Figure 6:**
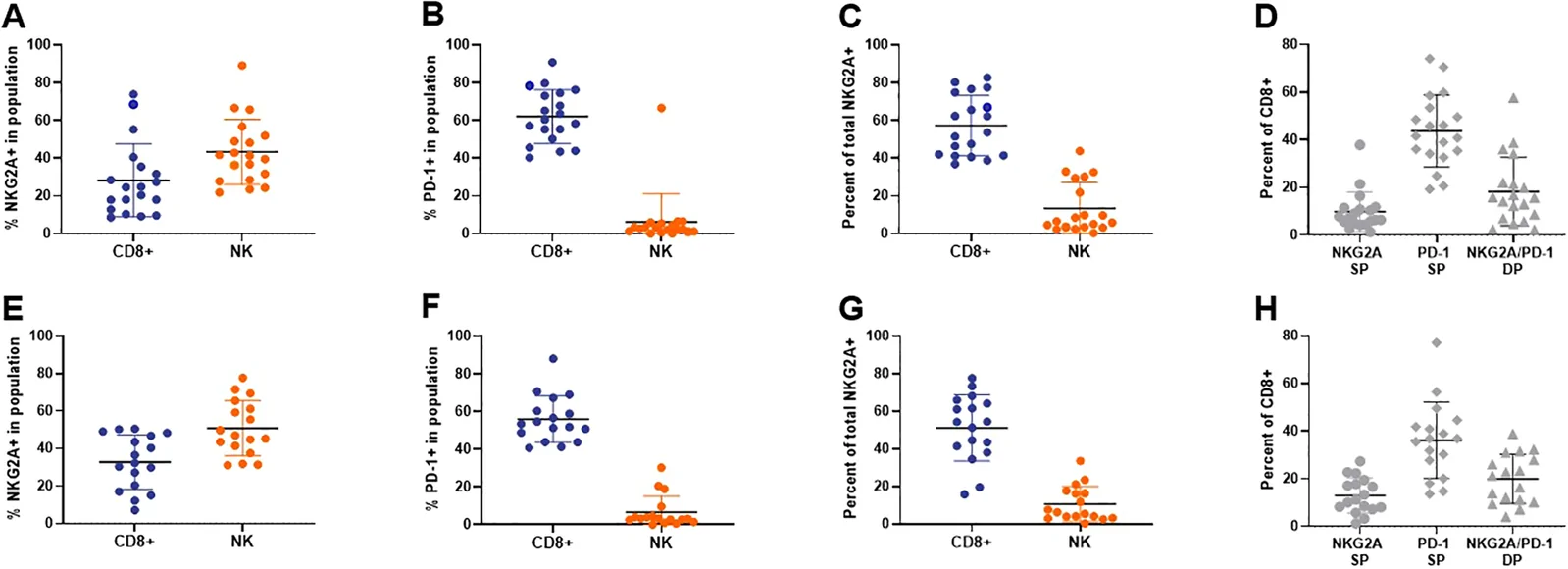
NKG2A expression in non-small cell lung cancer and gastric cancer patient samples. **(A)** Frequencies of CD8+ T cells and NK cells isolated from newly diagnosed NSCLC patients expressing NKG2A **(A)** or PD-1 **(B)** on their surface as determined by flow cytometry. **(C)** Respective proportions of CD8+ T cells and NK cells within the total NKG2A+ population of NSCLC TILs. **(D)** Percentage of NSCLC patients CD8+ TILs single positive for NKG2A or PD-1, as indicated, or double positive for both markers. Frequencies of CD8+ T cells and NK cells isolated from newly diagnosed gastric cancer patients expressing NKG2A **(E)** or PD-1 **(F)** on their surface as determined by flow cytometry. **(G)** Respective proportions of CD8+ T cells and NK cells within the total NKG2A+ population of gastric cancer TILs. **(H)** Percentage of gastric cancer patients CD8+ TILs single positive for NKG2A or PD-1, as indicated, or double positive for both markers. CD8+ T cells and NK cells were identified by gating on Epcam-/CD45+/CD3+/CD8+ cells and on Epcam-/CD45+/CD3-/CD56+, respectively. Total NKG2A+ cells were identified by gating on Epcam-/CD45+/NKG2A+ cells. Average values were: **(A)** 28.17% NKG2A+ for CD8+ T cells and 43.36% for NK cells; **(B)** 62.1% PD-1+ for CD8+ T cells and 6.24% for NK cells; **(C)** 57.35% for CD8+ T cells and 13.49% for NK cells; **(D)** 9.84% for NKG2A SP, 18.33% for DP, and 43.77% for PD-1 SP; **(E)** 32.76% for CD8+ T cells and 50.83% for NK cells; **(F)** 56.01% for CD8+ T cells and 6.62% for NK cells; **(G)** 51.19% for CD8+ T cells and 10.63% for NK cells; **(H)** 12.88% for NKG2A SP, 19.88% for DP, and 36.13% for PD-1 SP. The primary antibodies used were: CD3, CD4, CD8, CD16, CD39, CD45, CD56, CD73, EpCAM, HLA-E, LILRB1, NKG2A, PD-1, PD-L1, TCR γδ, and TIM-3. DP, double positive; hNKG2A, human natural killer group 2 family of receptor A; NK, natural killer; NSCLC, non-small cell lung cancer; PD-1, programmed cell death protein 1; SP, single positive; TIL, tumour-infiltrating lymphocyte.

## Discussion

We hereby report on the generation, selection, and functional characterization of the S095029 clinical candidate currently in dose expansion studies combined with PD-1 blocking agents.

S095029 was obtained from a broad repertoire of fully human antibodies triaged to selectively bind human NKG2A and minimise cross-reactivity with related NKG2x family members. The early inclusion of a functional assay as part of the NKG2A-binding profiling, in addition to disruption of the NKG2A/HLA-E interaction, ensured the selection of candidates with the desired biological characteristics. *In vitro* killing and cytokine release experiments demonstrated that S095029 more strongly enhanced γδ T cell activation as well as HLA-mismatch-induced and ADCC-mediated NK cell functions through NKG2A blockade than clinical-stage benchmark mAbs monalizumab and BMS986315. *In vivo* investigations showed that S095029 in combination with PD-1 blockade efficiently inhibited tumour growth in syngeneic mouse models. These results demonstrate an excellent efficacy profile in multiple immune cell types for S095029 and support its further clinical development in combination with PD-1 blockade.

Interestingly, when assessing the effect of NKG2A blockade on a broad panel of effector functions, we observed that the addition of S095029 to NK/tumour cell co-cultures differentially affected the secretion of soluble factors by NK cells. Specifically, MIP-1α and MIP-1β were more strongly upregulated than other secreted molecules. These two chemokines were also strongly upregulated in γδ-T cell activation assays in the presence of S095029 (data not shown). This suggests that NKG2A/HLA-E-mediated suppression may not affect all effector functions equally and that antibody blockade of this pathway could exert a qualitatively distinct stimulatory effect in immune cells susceptible to NKG2A suppression. In addition, the preferential modulation of MIP-1α and MIP-1β may provide useful exploratory pharmacodynamic markers to monitor NKG2A modulation in the clinic.

Like most NKG2A-targeting mAbs, S095029 does not cross-react with mouse NKG2A, limiting the possibilities for *in vivo* studies. However, we were able to test our clinical candidate in syngeneic tumour models of transgenic mice expressing human NKG2A and CD94. Whilst S095029 showed limited efficacy as a single agent in two syngeneic models, dual NKG2A/PD-1 blockade with the mouse PD-1 cross-reactive and clinically validated mAb SYM021 (35, 36) improved on anti-PD-1 monotherapy tumour growth inhibition. The anti-tumour potential of S095029, as a single agent or in combination with PD-1 blockade, was not affected by NK cell depletion. In contrast, CD8+ T cell depletion greatly reduced the therapeutic effects, highlighting the potential of NKG2A blockade in modulating the anti-tumour potential of CD8+ T cells. In addition, blocking the NKG2A-HLA-E pathway using S095029 combined with PD-L1-targeting avelumab in CD34-humanised mice engrafted with MDA-MB-231 breast adenocarcinoma tumour cells more strongly inhibited tumour growth (**Supplementary Figure 10**). Nevertheless, NKG2A blockade effectively harnessed the anti-tumour activity of NK cells as demonstrated in the experimental lung metastasis B16F10/HLA-E mouse model, where S095029 monotherapy significantly delayed lung tumour establishment in an NK cell-dependent manner. Even though the anti-tumour effects of combinatorial PD-1/NKG2A blockade appeared dominated by CD8+ T-cells, NK cells likely played a compensatory role contributing to the residual efficacy. The degree of NK cells contribution was variable, as evidenced by the differing single treatment efficacies of S095029 in various experiments with the same tumour model (**Figures 5B, D**). These distinct outcomes suggest that some variables, such as the basal level of NK cell infiltration during the early stages of tumour growth, may influence the therapeutic response and tumour growth rate. Consequently, a higher initial NK cell presence could potentially lead to more effective early tumour control and contribute to the observed compensatory mechanisms in CD8+ T cell depleted settings.

Phenotyping studies that evaluated clinically relevant NKG2A expression on cells obtained from dissociated gastric cancer and NSCLC, which are indications for which S095029 is currently being developed, indicated that NKG2A was frequently expressed on the surface of CD8+ T cells and NK cells within patient neoplastic tissues. An average of approximately 30% of CD8+ T cells were positive for NKG2A in both indications, albeit with high inter-patient variability. Importantly, CD8+ T cells constituted the largest population (between 40% and 80%) of NKG2A-expressing cells at the tumour site. CD8+ TILs are believed to be the primary population imparting the efficacy of PD-1 blockade, and baseline expression of both PD-1 and NKG2A should thus constitute a favourable setting for the combination of anti-PD-(L)1 with S095029, provided that sufficient intra-tumoural exposure can be achieved for both agents. These results are in line with previous phenotypic observations in solid tumours and expand on available information regarding ICP expression in these indications (15, 22). Interestingly, a substantial fraction of CD8+/NKG2A+ cells (approximately 30% on average) did not co-express PD-1, indicating that NKG2A blockade might help mobilise a CD8+ T cell population distinct from PD-1+ cells.

HLA-E tumour expression levels are also likely to influence the clinical efficacy of NKG2A-blocking antibodies. Whether baseline HLA-E expression constitutes a predictive biomarker of response is currently unknown but the presence of HLA-E at the tumour site has been documented in multiple studies (14, 26–28). In addition, IFN-γ promotes the expression of HLA-E and of its murine ortholog Qa-1^b^ (37, 38), which has been implicated as a resistance mechanism to PD-1 blockade in mouse models (39). It is thus conceivable that the dynamic interplay between immune checkpoint blockade, subsequent IFN-γ secretion and the induction of suppressive mechanisms such as HLA-E could further sensitise the tumour milieu to combinatorial PD-(L)1 and NKG2A blockade. The elucidation of these complex phenomena and their application as potential biomarkers will necessitate thorough translational investigations from clinical samples.

Promising results showing the clinical benefit of dual NKG2A/PD-L1 therapy administered to stage III NSCLC patients, either post-chemo-radiotherapy for unresectable tumours or in a neo-adjuvant setting, were recently published from the COAST and NeoCOAST studies (30, 31). These provide an encouraging clinical proof of concept for combining PD-1/PD-L1 and NKG2A targeting mAbs in disease settings responsive to immunotherapy. S095029 is currently being tested in first line in combination with standard-of-care cemiplimab in NSCLC patients with advanced, non-resectable PD-L1-high tumours as part of a platform trial also assessing the anti-TIM3 S095018 or the anti-CD73 S095024 (40) antibodies, each in combination with cemiplimab (41, 42). In a separate clinical study S095029 is combined to pembrolizumab in microsatellite instability-high gastric cancer patients. PD-1 blockade monotherapy objective response rates with these agents in similar settings are in the range of 40%-55% for microsatellite instability-high gastric cancer (43, 44) and 40-45% for NSCLC (45–47), suggesting potential scope for improvement with an adequate combination agent. Our translational results documenting the expression of NKG2A on TILs from these two indications support the notion that NKG2A blockade may provide additional clinical benefit to patients when it is combined with anti-PD(L)1 therapies.

The clinical safety of S095029 was recently established in a phase 1 dose escalation study, both in monotherapy and in combination with the SYM021 anti-PD-1 mAb (32). This was in line with our non-clinical safety assessment, which showed no alerts (data not shown) and where S095029 did not induce ADCC or complement-dependent cytotoxicity on cells expressing physiological levels of NKG2A *in vitro*. This latter point constitutes a differentiating aspect of attenuated Fc-receptor binding antibody formats such as IgG1-LALA compared with Fc-receptor competent mAbs and might affect antibody function in the clinic.

In summary, the preclinical efficacy results of S095029, alone or in combination with PD-1 blockade, together with new translational data documenting the expression of both PD-1 and NKG2A in NSCLC and gastric cancer patient tumours, support the clinical evaluation of a novel NKG2A blocking agent showing a favourable preclinical efficacy profile.

## Data Availability

The raw data supporting the conclusions of this article will be made available by the authors, without undue reservation.
