# Supplementary material for: Preclinical characterization of S095029, a novel clinical-stage Fc-attenuated NKG2A-blocking antibody for cancer immunotherapy
Source: Front Oncol. 2026 Sep 23;16:1924010. doi: 10.3389/fonc.2026.1924010 (PMC13643682; doi:10.3389/fonc.2026.1924010)
Supplement: Supplementary file 1 [file SupplementaryFile1.docx]

# Supplementary Information for “Preclinical characterization of S095029, a novel clinical-stage Fc-attenuated NKG2A-blocking antibody for cancer immunotherapy”

# Supplementary Tables

| **Cell line name** | **Supplier** | **Reference** |
| --- | --- | --- |
| A375 | ATCC | CRL-1619 |
| K562 | ATCC | CCL-243 |
| HEK293 | ATCC | CRL-1573 |
| HEK293T | Abcam | 255449 |
| A431 | ATCC | CRL-1555 |
| HT-29 | ATCC | HTB-38 |
| CCRF-CEM | ATCC | CCL-119 |
| A253 | ATCC | HTB-41 |
| Detroit 562 | ATCC | CCL-138 |
| CAL-120 | DSMZ | ACC 459 |
| FaDu | ATCC | HTB-43 |
| JIMT-1 | DSMZ | ACC589 |
| SKOV-3 | ATCC | HTB-77 |
| BxPC3 | ATCC | CRL-1687 |
| A549 | ATCC | CCL-185 |
| SUDHL-6 | ATCC | CRL-2959 |

**Table S1:** Cell lines used in this study.

| **Antigen** | **Supplier** | **Reference** |
| --- | --- | --- |
| CD39 (human) | BD | 567508 |
| CD4 (human) | BD | 612936 |
| PD1 (human) | BD | 612791 |
| PDL1 (human) | BD | 563738 |
| CD73 (human) | BD | 563198 |
| HLA-E (human) | BD | 751743 |
| TIM3 (human) | Biolegend | 345028 |
| CD16 (human) | BD | 563690 |
| TCR gd (pan) (human) | Miltenyi | 130-113-511 |
| CD8 (human) | Biolegend | 344708 |
| CD56 (human) | BioLegend | 362508 |
| EpCAM (human) | Miltenyi | 130-111-001 |
| LILRB1 (human) | Biolegend | 333719 |
| NKG2a (human) | Miltenyi | 130-128-577 |
| NKG2a (human) | Beckman Coulter | A60797 |
| CD3 (human) | Biolegend | 300318 |
| CD45 (human) | BD | 742020 |
| CD3 (mouse) | Biolegend | 344804 |
| CD335 (mouse) | BD Pharmingen | 557991 |
| CD56 (mouse) | Biolegend | 318322 |
| CD45 (mouse) | Biolegend | 304016 |
| CD8 (mouse) | BD Pharmingen | 560179 |
| CD16 (mouse) | Biolegend | 302038 |
| HLA-E pentamer | Proimmune | F712-2C_E |

**Table S2:** Flow cytometry antibodies and reagents used in this study.

| Variables | N | % |
| --- | --- | --- |
| **Diagnosis** |  |  |
| DLBCL | 5 | 62.5 |
| FL | 3 | 37.5 |
| **Age** |  |  |
| ≤60 y/o | 4 | 50 |
| >60 y/o | 4 | 50 |
| **Sex** |  |  |
| Female | 6 | 75 |
| Male | 2 | 25 |
| **Stage** |  |  |
| I-II | 3 | 37.5 |
| III | 1 | 12.5 |
| IV | 4 | 50 |
| **Cell of Origin (DLBCL)** | | |
| GCB | 1 | 20 |
| non-GCB | 4 | 80 |
| **Relapse status at biopsy** | | |
| Non-relapse | 6 | 75 |
| Relapse | 2 | 25 |

**Table S3:** Patient characteristics of diffuse large B-cell lymphoma and follicular lymphoma samples included in this study. Abbreviations: DLBCL, diffuse large B-Cell lymphoma; FL, follicular lymphoma; GCB, germinal center B-cell sub-type of diffuse large B-cell lymphoma and follicular lymphoma.

| DTC ID | Patient ID | Diagnosis | Tumour location | Overall Clinical Stage | Overall Treatment Status | Gender | Patient age at collection |
| --- | --- | --- | --- | --- | --- | --- | --- |
| NSCLC#1 | 130957490 | Lung Cancer, Squamous Cell Carcinoma | Left Lung | II-B | Pre Tx | Male | 65 |
| NSCLC#2 | 130953969 | Lung Cancer, Squamous Cell Carcinoma | Left lung | II-B | Pre Tx | Female | 68 |
| NSCLC#3 | 130953970 | Lung Cancer, Squamous Cell Carcinoma | Left lung | II-B | Pre Tx | Male | 67 |
| NSCLC#4 | 130950786 | Lung Cancer, Adenocarcinoma | Left lung | I-B | Pre Tx | Female | 70 |
| NSCLC#5 | 130959438 | Lung Cancer, Adenocarcinoma | Lymph node | IV | Pre Tx | Female | 47 |
| NSCLC#6 | 130957492 | Lung Cancer, Adenocarcinoma | Left Lung | III-B | Pre Tx | Male | 67 |
| NSCLC#7 | 130959436 | Lung Cancer, Adenocarcinoma | Right lung | III-B | Pre Tx | Male | 76 |
| NSCLC#8 | 130942084 | Lung Cancer, Squamous Cell Carcinoma | Upper lobe of the right lung | I-B | Pre Tx | Male | 67 |
| NSCLC#9 | 200009106 | Lung Cancer, Squamous Cell Carcinoma | Lung | II-B | Pre Tx | Male | 63 |
| NSCLC#10 | 130956857 | Lung Cancer, Adenocarcinoma | Right lung | I-B | Pre Tx | Male | 63 |
| NSCLC#11 | 200036616 | Lung Cancer, Squamous Cell Carcinoma | Lung | I-A | Pre Tx | Female | 67 |
| NSCLC#12 | 200015347 | Lung Cancer, Adenocarcinoma | Right lung | II-A | Pre Tx | Male | 64 |
| NSCLC#13 | 200020866 | Lung Cancer, Squamous Cell Carcinoma | Right lung - upper lobe | I-A | Pre Tx | Female | 75 |
| NSCLC#14 | 200036616 | Lung Cancer, Adenocarcinoma | Lung | I-A | Pre Tx | Male | 64 |
| NSCLC#15 | 200019439 | Lung Cancer, Squamous Cell Carcinoma | N/A | III-B | Pre Tx | Male | 63 |
| NSCLC#16 | 200009386 | Lung Cancer | Lower left lobe | III-B | Pre Tx | Male | 64 |
| NSCLC#17 | 130807758 | Lung Cancer, Adenocarcinoma | Left Lung | III-B | Pre Tx | Male | 76 |
| NSCLC#18 | 122523317 | Lung Cancer | Upper lobe of lung (Left) | II-B | Pre Tx | Unknown (Reviewed) | 61 |
| NSCLC#19 | 122500955 | Lung Cancer, Squamous Cell Carcinoma | Upper lobe of lung | III-A | Pre Tx | Male | 62 |
| GC#1 | 130950651 | Gastric Cancer, Adenocarcinoma | Stomach | III-B | Pre Tx | Male | 62 |
| GC#2 | 130958989 | Gastric Cancer, Adenocarcinoma | Stomach | IV | Pre Tx | Male | 74 |
| GC#3 | 200020481 | Gastric Cancer, Adenocarcinoma | Stomach | Unknown (Reviewed) | Pre Tx | Male | 74 |
| GC#4 | 130950650 | Gastric Cancer, Adenocarcinoma | Stomach | III-C | Pre Tx | Female | 51 |
| GC#5 | 130950648 | Gastric Cancer, Adenocarcinoma | Stomach | III-B | Pre Tx | Male | 65 |
| GC#6 | 110043010 | Gastric Cancer, Adenocarcinoma | Stomach, NOS | III-B | Pre Tx | Male | 60 |
| GC#7 | 110039979 | Gastric Cancer, NOS | Stomach | II-B | Pre Tx | Male | 59 |
| GC#8 | 200000842 | Gastric Cancer, Adenocarcinoma | Antral part of the stomach | II-A | Pre Tx | Male | 77 |
| GC#9 | 110039887 | Gastric Cancer, Adenocarcinoma | N/A | I-B | Pre Tx | Male | 66 |
| GC#10 | 200036808 | Gastric Cancer, Adenocarcinoma | Stomach | IV | Pre Tx | Male | 73 |
| GC#11 | 130776717 | Gastric Cancer, Adenocarcinoma | Stomach | Unknown (Reviewed) | Pre Tx | Male | 57 |
| GC#12 | 200013667 | Gastric Cancer, Adenocarcinoma | Stomach | Unknown (Reviewed) | Pre Tx | Female | 71 |
| GC#13 | 200018344 | Gastric Cancer, Adenocarcinoma | N/A | III-B | Pre Tx | Male | 40 |
| GC#14 | 110047126 | Gastric Cancer, NOS | Stomach, NOS | III-B | Pre Tx | Male | 61 |
| GC#15 | 200036806 | Gastric Cancer, Adenocarcinoma | Stomach | Unknown (Reviewed) | Pre Tx | Male | 77 |
| GC#16 | 200020257 | Gastric Cancer, Adenocarcinoma | Gastric | Unknown (Reviewed) | Pre Tx | Male | 84 |
| GC#17 | 120746456 | Gastric Cancer, Adenocarcinoma | Stomach, greater curvature | III-A | Pre Tx | Male | 53 |

**Table S4:** Data on dissociated tumour and tissue cells from non-small cell lung cancer and gastric cancer patients. Abbreviations: DTC, dissociated tumour and tissue cells; ID, identifier; GC, gastric cancer; NSCLC, non-small cell lung cancer; pre Tx, pre-treatment.

# Supplementary Figures

**Figure S1. Binding of selected antibodies to human and cynomolgus NKG2A and NKG2C.** Antibodies were prepared in three-fold serial dilutions from 3 µg/ml and tested for binding to CHO-S cells transiently transfected with human NKG2A/CD94, cNKG2A/cCD94, hNKG2C/hCD94, and cNKG2C/cCD94. A secondary AF647 fluorochrome-labelled a-human IgG(H+L) was used as detection antibody. The lead candidate monoclonal antibody 24208 had the lowest binding to hNKG2C/hCD94. Only monalizumab showed cNKG2C/cCD94 binding. Abbreviations: c, cynomolgus; CHO-S, Chinese hamster ovary; human (h); IgG1, immunoglobulin G1; NKG2A; natural killer group 2 family of receptor A; NKG2C, natural killer group 2 member C.

**Figure S2. Blocking of HLA-E pentamer by selected antibodies.** Antibodies were prepared in three-fold serial dilutions from 30µg/ml and tested for blocking the human leukocyte antigen-E (HLA-E) binding to CHO-S cells transiently transfected with human hNKG2A)/cCD94, cNKG2A/cCD94, hNKG2C/hCD94 and cNKG2C/cCD94. Antibodies were added to cells and incubated for 20 minutes at 4˚C. After washing, HLA-E pentamer was added to the cells at a 1:50 final dilution and incubated for 20 minutes at 4˚C in the dark. Cells were acquired on an iQue screener flow cytometer to measure binding. Antibody concentrations ranged from 30 µg/ml to 14 ng/ml (three-fold dilutions). The graphs show binding of HLA-E in presence of different a-NKG2A antibodies. All antibodies block binding of HLA-E to hNKG2A/hCD94, whereas only monalizumab and 23686 block the cNKG2A/cCD94 and cNKG2C/cCD94 binding with high potency. HLA-E binding to hNKG2C/hCD94 is only blocked fully by 23686, and with lower potency by 23566. Abbreviations: c, cynomolgus; CHO-S, Chinese hamster ovary; ctrl, control; human (h); IgG1, immunoglobulin G1; NKG2A; natural killer group 2 family of receptor A; NKG2C, natural killer group 2 member C.

**Figure S3. Kinetic measurements of the interactions between anti-NKG2A monoclonal antibodies and CD94/NKG2x heterodimers with five different agonist pMHCI ligands.** Kinetic binding analysis of the purified anti-NKG2A antibodies was performed by surface plasmon resonance (SPR) on an IBIS MX-96 biosensor (IBIS, Netherlands). Human his-tagged and flag-tagged hNKG2A/hCD94 and his-tagged hCD94 homodimer, used for first pass-affinity measurements, were from Novo Protein. Human and cynomolgus NKG2A/CD94 and NKG2C/CD94 heterodimers were produced in-house. Binding kinetics were measured under monovalent antigen conditions by immobilizing anti-NKG2A antibodies and keeping the monovalent antigen in solution. The lowest possible anti-NKG2A antibody density was applied to prevent non-specific binding and mass transport limitation. For measuring antibody kinetics, anti-NKG2A antibodies were adjusted to a concentration of 0.4 µg/ml and captured on an anti-human IgG Fc sensor (SensEye, Netherlands) surface. Anti-NKG2A antibodies were tested for binding to antigens at 100 nM and three-fold dilutions thereof to 0.14 nM. After antigen titrations, the surface was regenerated with 100 mM phosphoric acid, pH 3. An association and dissociation time of 15 minutes was employed for the kinetic screening. The recorded binding responses were fitted to a simple Langmuir 1:1 binding model for calculation of the on-rate (k_on_ or k_a_), off-rate (k_off_ or k_d_), and affinity (K_D_) constants.

S095029 & hNKG2A

S095029 & hNKG2C

Mona & hNKG2A

Mona & hNKG2C

Abbreviations: hNKG2A, natural killer group 2 family of receptor A; NKG2C, natural killer group 2 member C; mona, monalizumab; pMHCI, peptide-major histocompatibility class I.

**Figure S4. Cytokine upregulation elicited by NKG2A blockade with S095029 or a BMS986315 analogue.** Co-cultures of expanded primary natural killer (NK) cells and the A375/human leukocyte antigen-E (HLA-E) target cell line overexpressing the GD2 and GD3 enzymes in presence of increasing anti-NKG2A antibody concentration (as indicated in X). The experiment was performed on expanded NK cells isolated from four donors (top to bottom). IFN-g, MIP-1a, and MIP-1b concentrations were measured from cell supernatants after 48 hours. X values were then transformed logarithmically (X = LogX), and a nonlinear regression (curve fit) analysis was applied with a constant equal to the highest concentration measured as top constraint. Duplicate cytokine concentrations are shown for each antibody concentration. Abbreviations: Ab, antibody; IFN, interferon; MIP, macrophage inflammatory protein; NKG2A, natural killer group 2 family of receptor A.

**Figure S5. Cytokine upregulation elicited by NKG2A blockade with S095029 or a monalizumab analogue.** Co-cultures of expanded primary natural killer (NK) cells and the A375/human leukocyte antigen-E (HLA-E) target cell line overexpressing the GD2 and GD3 enzymes in presence of increasing anti-NKG2A antibody concentrations (as indicated in X). The experiment was performed on expanded NK cells isolated from three donors (top to bottom). IFN-g, MIP-1a and MIP-1b concentrations were measured from cell supernatants after 48 hours. X values were then transformed logarithmically (X = LogX), and a nonlinear regression (curve fit) analysis was applied with a constant equal to the highest concentration measured as top constraint. Duplicate cytokine concentrations are shown for each antibody concentrations. Abbreviations: Ab, antibody; IFN, interferon; MIP, macrophage inflammatory protein; NKG2A, natural killer group 2 family of receptor A.

**A**

**B**

**C**

**D**

**E**

**F**

**G**

**H**

**I**

**J**

**K**

**Figure S6. Enhancement of ADCC-induced (FcR-mediated) NK cell activation with NKG2A-blocking antibodies in co-cultures with tumour cells.** (**A**) S95029-mediated enhancement of the cytotoxic activity induced by cetuximab on the epidermal growth factor receptor (EGFR)-expressing cell line FaDu. 10 µg/ml S09529 or IgG1-LALA were added to primary NK cells co-cultured with FaDu cells in presence of increasing concentrations of cetuximab as indicated on the x-axis. (**B**) EC_50_ and maximum activity (**C**) values of A375/HLA-E+ target cell dinutuximab-mediated ADCC enhancement by S95029, BMS986315, or monalizumab antibody analogues using *ex vivo* expanded primary NK cells isolated from healthy donors. Each dot represents a donor. (**D**) EC_50_ and maximum activity (**E**) values of SKOV-3/HLA-E+ target cell trastuzumab-mediated ADCC enhancement by S95029, BMS986315, or monalizumab antibody analogues using *ex vivo* expanded primary NK cells isolated from healthy donors. S095029-mediated increase in IFN-g secretion (**F**) or cell-surface CD137 expression (**G**) by primary NK cells co-cultured with A431 target cells without treatment or in the presence of cetuximab alone or with S095029 at 10 µg/ml. (Cetux + S = cetuximab + S095029; Cetux + ctrl = cetuximab + IgG1-LALA 10 µg/ml). (**H-K**) Increase in MIP-1b secretion by S095029 (66.66 nM) in co-cultures of primary NK cells with cell lines overexpressing HLA-E, as indicated, in presence of trastuzumab 0.1 µg/ml or IgG isotype control. Wilcoxon signed rank test was applied for two-group comparisons of non-paired non-parametric data: ns (not significant) >0.05, *<0.05, **<0.01). Statistical analyses were performed using the R software version 4.4.1. Abbreviations: Ab, antibody; ADCC, antibody-dependent cell-mediated cytotoxicity; EC_50_, half maximal effective concentration; HLA-E, human leukocyte antigen-E; IFN, interferon; IgG1, immunoglobulin G1; MIP, macrophage inflammatory protein; mona, monalizumab; NK, natural killer; NKG2A, natural killer group 2 family of receptor A; untr., …; WT, wild type.

**Figure S7 Cytokine upregulation elicited by NKG2A blockade with S095029 or a BMS986315 analogue in the presence of dinutuximab.** Co-cultures of expanded primary natural killer (NK) cells and the A375/HLA-E target cell line overexpressing the GD2 and GD3 enzymes in the presence of increasing anti-NKG2A antibody concentrations (as indicated in X). The final concentration of dinutuximab was 1 µg/ml. The experiment was performed on expanded NK cells isolated from four donors (top to bottom). IFN-g, MIP-1a, and MIP-1b concentrations were measured from cell supernatants after 48 hours. X values were then transformed logarithmically (X = LogX), and a nonlinear regression (curve fit) analysis was applied with a constant equal to the highest concentration measured as top constraint. Duplicate cytokine concentrations are shown for each antibody concentration. Abbreviations: Ab, antibody; IFN, interferon; IgG1, immunoglobulin G1; MIP, macrophage inflammatory protein; NKG2A, natural killer group 2 family of receptor A.

**Figure S8. Cytokine upregulation elicited by NKG2A blockade with S095029 or a monalizumab analogue in the presence of dinutuximab.** Co-cultures of expanded primary NK cells and A375/HLA-E target cell line overexpressing the GD2 and GD3 enzymes in the presence of increasing NKG2A antibody concentrations (as indicated in X). The final concentration of dinutuximab was 1 µg/ml. The experiment was performed on expanded natural killer (NK) cells isolated from four donors (top to bottom). IFN-g, MIP-1a, and MIP-1b concentrations were measured from cell supernatants after 48 hours. X values were then transformed logarithmically (X = LogX), and a nonlinear regression (curve fit) analysis was applied with a constant equal to the highest concentration measured as top constraint. Duplicate cytokine concentrations are shown for each antibody concentration. Abbreviations: Ab, antibody; IFN, interferon; IgG1, immunoglobulin G1; MIP, macrophage inflammatory protein; NKG2A, natural killer group 2 family of receptor A.

**Figure S9**. Individual tumor growth curves of MCA205 and MC38-HLAE tumor studies in hNKG2A/hCD94 KII mice. Arrows indicate treatment start; red lines show mean tumor growth. Depletion of NK and CD8 cells was verified by flow cytometer analysis of whole blood (bottom plots). NK cells were gated as CD3-CD335+ cells, and CD8 cells as CD45+CD8+ cells. Abbreviations: h, human; HLAE, human leukocyte antigen-E; NK, natural killer; NKG2A, natural killer group 2 family of receptor A; PD1, programmed cell death protein 1.

MCA205-HLAE

MC38-HLAE +/- NK depletion

MC38-HLAE +/- CD8 depletion

**Figure S10.** CD34-humanized mice were engrafted with 10^7^ MDA-MB-231 breast adenocarcinoma tumor cells and treated three times weekly intraperitoneally with S095029 in combination with PD-L1 monoclonal antibody (mAb) avelumab (human immunoglobulin G1). Individual tumor growth curves are shown in the right panels; red lines show mean tumor growth. Abbreviations: PDL1, programmed death-ligand 1. At an average tumour size of 50 mm^3^, mice were boosted by hydrodynamic gene delivery whereby a volume corresponding to 8% of body weight containing 6.25 µg/mL hIL15 plasmid DNA was injected i.v. within 10 seconds. Treatment was initiated at an average tumour volume of 65 mm^3^; mice were randomized into treatment groups based on immune cell phenotype, tumour volume and CD34 donor origin, and were treated three times weekly by intraperitoneal (i.p.) injection of vehicle buffer, S095029 (10 mg/kg), anti-PDL1 (10 mg/kg SYM021 +ref 33 34), or a combination of S095029 and anti-PDL1 (n=10/group, approximately 20% of the total number of mice included in the experiment were removed at randomization based on low degree of humanization or outlier tumour volumes ).

MDA-MB-231 in CD34 humanized mice
